# Lockdown During COVID-19 and the Increase of Frailty in People With Neurological Conditions

**DOI:** 10.3389/fneur.2020.604299

**Published:** 2020-11-16

**Authors:** Paulo H. S. Pelicioni, Jennifer S. Schulz-Moore, Leigh Hale, Colleen G. Canning, Stephen R. Lord

**Affiliations:** ^1^Neuroscience Research Australia, University of New South Wales, Sydney, NSW, Australia; ^2^Faculty of Law, University of New South Wales, Sydney, NSW, Australia; ^3^School of Public Health and Community Medicine, University of New South Wales, Sydney, NSW, Australia; ^4^School of Physiotherapy, University of Otago, Dunedin, New Zealand; ^5^Discipline of Physiotherapy, Faculty of Medicine and Health, The University of Sydney, Sydney, NSW, Australia

**Keywords:** lockdown, neurological conditions, COVID-19, frailty, falls, physical inactivity, health policy

## Abstract

Governments around the globe have introduced quarantine, lockdown, and mandatory isolation to slow the transmission of COVID-19. These public health and policy measures aim to protect the public and vulnerable people. This perspective paper argues that the impacts of lockdown (such as social disconnection, reduced exercise, and fewer physiotherapy treatments) may be amplified for people with neurological conditions with subsequent increases in frailty. The paper outlines why this may occur, and explores how adverse impacts for these vulnerable populations may be minimized through strategies such as telehealth, exercise programs, and health policies.

## Introduction

In March 2020, the World Health Organization declared that severe acute respiratory syndrome coronavirus 2 (SARS-CoV-2) infection, or coronavirus disease (COVID-19), is a pandemic. To reduce the spread of COVID-19 infection, most governments around the world adopted public health policies such as lockdown, isolation, and quarantine. Quarantine restricts the movement of people who may have been exposed to an infectious disease, but who are not yet symptomatic. Isolation involves separation of those who are infected. In Australia, pursuant to section 4(1) of *The Quarantine Act 1908* (Cth), quarantine includes “the examination, exclusion, detention, observation, segregation, isolation, protection, treatment and regulation of vessels, installations, human beings, plants or other goods or things.” In federal jurisdictions such as Australia, the “lockdown” rules differ according to individual states and territories. Each level of lockdown is comprised of varying degrees of restrictions (such as whether non-essential services are able to operate) and lockdowns may be staged. The outcomes of these government measures may include social isolation and reduced access to health services.

Government orders such as quarantine, lockdown and mandatory isolation are primarily adopted to protect people's health, especially older people and those with chronic lung, liver, renal or heart disease, diabetes, obesity, cancer in the past 12 months, neurological conditions such as stroke or dementia and poorly controlled hypertension. Vulnerable populations and older people have particularly poor prognoses associated with COVID-19 because of complications such as acute respiratory distress syndrome and pneumonia (1, 2). With the death of over 1 million people worldwide (at the time of writing in mid-October 2020), lockdown is a crucial measure to reduce the likelihood of death among vulnerable populations (3, 4). However, social disconnection due to lockdown during COVID-19 may increase frailty in people with neurological conditions (5, 6). For example, Helmich and Bloem (5) recently argued that there are several “hidden sorrows” and “highly disconcerting consequences” of lockdown, such as the deleterious impact on people with Parkinson's disease (PD) because of their reduction in physical activity and disengagement with physiotherapists (5).

The objectives of this perspective paper are to outline why lockdown can have negative physical, cognitive, and mental health outcomes for people with neurological conditions as well as highlight the potential of telehealth and exercise regimes to minimize such adverse health outcomes. It concludes with a discussion of a perennial problem in public health law and policy: the tension between an individual's rights and public health.

## What Are the Impacts of Lockdown on People With Neurological Conditions?

The adoption of lockdown measures has saved many lives by reducing the spread of COVID-19. However, as argued elsewhere, such measures may have been a “two-edged sword” (1). While lockdown has been essential, governments and health systems have not been well-prepared to manage the consequences of social disconnection caused by this policy (2, 3). It is well-documented that social disconnection has negative health impacts (7, 8), and these negative impacts are magnified for people (such as those with neurological conditions) who need extensive care, physiotherapy, and regular activity to maintain their well-being and health. Although essential services have remained open, traditional face-to-face therapies have been modified, reduced and, sometimes, canceled. In contrast, there is evidence that physical exercise protocols for healthy people have only been slightly affected by the pandemic (2) with these individuals maintaining their exercise routines, often through the use of technology (3, 4).

However, health systems (burdened with the pandemic) have struggled to meet the needs of vulnerable people, especially people with neurological disorders (9). For example, it is known that stroke survivors need physiotherapy and stimulation upon discharge from inpatient rehabilitation, and such approaches result in better outcomes (10). People with progressive conditions such as PD and Multiple Sclerosis (MS) need maintained exercises to reduce the decline of functional capacity associated with the progressive nature of these pathologies (11–13). Exercise cessation in these groups can reduce cardiorespiratory fitness, muscle strength, muscle mass and impair cognition, leading to an “accelerated” loss of function and significantly reduced quality of life (14, 15). Consequently, a series of health and social issues are likely to arise in the forthcoming months and years (16).

## Lockdown and Declining Mental Health in People With Neurological Conditions

Lockdown during the COVID-19 pandemic has caused an increase in mental health issues such as depression and anxiety (6). Previous research has reported that lack of neighborhood engagement and social connection contribute to an increased risk of mental health disorders due to loneliness (7). Researchers have also found that social disconnection remains a strong risk factor for increased mortality in older people, even after adjusting for demographic and health factors (8).

This association may be amplified for people with neurological disorders as a consequence of the pandemic for three key reasons. First, mental health conditions, particularly anxiety, and depression, are more prevalent in people with PD, people with MS, and people with cognitive impairment and dementia (17–19). These conditions may worsen during a pandemic because of the loneliness, social disconnection, and the pandemic-related uncertainties.

Second, the mental health of people with neurological conditions may decline because of the additional stressors on caregivers caused by lockdown. Caregivers experiencing multiple stressors may be unable to provide adequate care to people with neurological conditions (20). In some countries, such as Brazil and New Zealand, caregivers are not considered essential workers, thus they have had to stop “caring” for their clients. These circumstances create a troubling loop of poor health outcomes for both caregivers and care recipients.

Third, the decrease in visitors to residential facilities due to lockdown may negatively impact mental health in people with neurological conditions (21, 22). Government public health policies have meant that residents in residential care facilities either had fewer, or no, visits from people outside of the facility (23). These impacts may be amplified for people with neurological conditions, many of whom already suffer from mental health illnesses.

## Falls—From a Cascade to a Loop Effect

Lockdown measures can lead to an increased risk of falls by multiple, inter-related means. First, lockdown reduces many people's physical activity levels and the negative health impacts of lack of exercise are well-documented (14, 24). Second, isolation at home can also lead to lack of exposure to sunshine, and as a result, Vitamin D deficiency (25). Consequently, the immune system can be compromised and infections can occur at a higher rate. Third, polypharmacy is a well-known risk factor for falls (26, 27), and people with neurological conditions may take more medications to cope with the negative consequences of lockdown. For example, lack of physical exercise in stroke survivors and people with MS may predispose them to joint and muscle stiffness, and increased pain, associated with an increase in pain medication usage.

Furthermore, during a pandemic, people with neurological disorders may be more inclined to stay indoors. Confined home environments can also hamper ambulation which can impair transfers and adaptive gait in people with balance and mobility impairments, as well as freezing of gait in people with PD, which can lead to falls (28–30). Declines in mental and physical health associated with sedentary behavior may increase fear of falling (31–33). Combined, these complications can result in a downward spiral adversely impacting health and increasing the likelihood of recurrent falls and serious injuries such as head trauma and fractures.

## Telehealth—a Solution for Addressing the Increased Frailty in People With Neurological Conditions During a Pandemic?

Telehealth has enormous potential for assisting people with therapies and exercise delivery (34), with many health providers now offering telehealth consultations and treatments for musculoskeletal injuries and physical exercises during COVID-19 (35, 36). Telehealth is becoming an important component of health care systems because it has the potential to address challenging issues such as access to health care and health inequalities (34). For example, telehealth has been used successfully with people living in remote areas, such as indigenous communities and rural populations (37, 38).

However, telehealth presents challenges. For example, it is not an affordable option for all patients and not all countries provide reliable internet services. In addition, using telehealth may be challenging for people with neurological disorders. Telehealth interventions involve the individual performing activities without face-to-face interaction with a therapist. During assessment and treatment without a therapist present, people with neurological conditions may present an increased risk of balance loss and, consequently, fall. Although there is some evidence that home-based protocols may be safe and suitable for individuals with mild to moderate impairments (39), previous studies have included at least one home visit to set up home-based exercises and associated technology, including virtual reality technology (40–44).

The risks are likely to be higher for individuals with more severe disease. For example, while minimally-supervised home-based exercise programs are effective in reducing falls in individuals with mild to moderate PD, they can lead to an increase in the proportion of fallers in individuals with more severe disease (45–47). This is likely due to previous multiple falls, freezing of gait, and cognitive impairment in those with more severe disease, who may require more targeted, supervised sessions to gain therapeutic benefit. Individuals with severe neurological disability or progressive or acute onset usually present with major balance impairment, or may not be able to stand without support. In a lockdown, a telehealth approach, with no supervised exercise, could be harmful to these individuals. Physiotherapy for individuals with severe neurological disorders requires careful assessment of motor, cognitive and emotional impairments. In some cases, patients are not able to perform their exercises without the therapist's assistance (48, 49).

To surmount these challenges, further research is needed to test the safety and feasibility of telehealth assessments and interventions for people with severe neurological disorders. There are information gaps around delivering physiotherapy programmes and rehabilitation for people with neurological disorders via telehealth; in particular, in relation to assessment and measurement, which are vital to appropriate delivery of rehabilitation. Telehealth is a healthcare delivery model which could improve patient care and decrease social disconnection, but validated evidence is needed to address known gaps and to correctly and effectively inform clinical practice (34).

## Running Against the Clock—How Can We Minimize the Accelerated Progression Potentially Caused by Lockdown?

Many neurological disorders (such as Alzheimer's disease, PD, MS) are progressive. The consequences of social disconnection due to lockdown (e.g., an exacerbation of inactive lifestyle and reduction in healthcare appointments) may increase the rate of progression of these neurological disorders. Therefore, with the relaxation in lockdown policies in some countries, it is time to implement clinical strategies to reduce the accelerated progression of neurological disorders. One clinical strategy, for people who are in the early stages of the disease, is multicomponent exercise. Jimenez-Pavol et al. (50), recently published an exercise recommendation for older people that may suit people in the early stages of neurological conditions. These recommendations need to be carefully tailored for individuals' conditions, symptoms, context, and preferences. For people in the more severe stages of their condition, a personalized approach is needed to identify whether new symptoms or complications have emerged since the commencement of lockdown. If therapists identify such symptoms or complications (e.g., freezing of gait in a person with PD), then specific evidence-based interventions should be initiated to address these problems.

Furthermore, physiotherapists should be considered “essential workers.” Widespread use of telehealth for initial screening of people with neurological conditions would help to stratify individuals, maximize outcomes and minimize risks. Thereafter, physiotherapists could provide periodic in-person visits with the use of personal protective equipment (PPE) and precautions, to ensure safety and progression of home-based exercises. For individuals who suffer from severe gait and balance impairments, prescription of assistive devices should be considered to optimize safety, as well as occupational therapy interventions based on safe mobility training (51). Other measures, such as the installation of bars in the shower or toilet, or the removal of environmental hazards, may help to prevent falls in the home setting.

As mentioned in the section of this paper entitled “What Are the Impacts of Lockdown on People with Neurological Conditions?,” caregivers may also be negatively impacted because of social disconnection during the pandemic. Therefore, exercises and therapies may also be offered to primary caregivers to improve their mental and physical health. By caring for the caregivers, delivery of optimal care for people with neurological disorders is more likely to be achieved (52).

## The Role of Public Health Law: Balancing the Patient's Rights With Public Health During a Pandemic

According to Gostin (53), public health law is the “study of the legal powers and duties of the state to assure the conditions for people to be healthy and the limitations on the power of the state to constrain the autonomy, privacy, liberty, proprietary, or other legally protected interests of individuals for the common good.” Public health laws offer essential tools for improving health and preventing illness and injury. Regulatory devices are some of the oldest and most significant tools available to public health policymakers and practitioners. The valuable role of law is evident in all of public health's greatest achievements (54). For example, the control of infectious diseases is supported by legal tools such as sanitary codes, drinking water standards, quarantine and isolation authority, building codes, pest control programmes, and inspection of food establishments (55).

Tension between policy options arose in the nineteenth and early twentieth centuries during infectious disease outbreaks and has re-emerged as health systems introduced public health laws and policies to address chronic conditions (53, 56). Infectious disease laws, introduced throughout the twentieth century, have provided extensive and coercive public health powers to prevent the spread of communicable disease. The essence of the tension is that policymakers and legislatures need to make difficult trade-offs when making determinations about the weight to give to the individual (and his/her health needs and rights), versus the public's health, or the collective good (57).

Laws authorizing the quarantine of people with certain infectious diseases are possible in jurisdictions which have legal traditions that permit limits to individual liberty to protect the common good. In doing so, there is a policy trade-off whereby other rights are given less weight. As we, and others (5, 6), have argued, the adverse impacts caused by lockdown (such as social disconnection and reduced exercise) may be magnified for people with neurological conditions. During COVID-19, policymakers have chosen to introduce measures such as lockdown to prioritize lives and the collective good. Policymakers also aimed to protect older people and vulnerable populations. However, as outlined above, these policies have been a “two-edged sword” for people with neurological conditions.

Given this outcome, this paper argues that policymakers and legislatures should consider amendments to regulations and policies to support interventions which could minimize the impact of lockdown, especially for vulnerable populations. This recalibration could be undertaken while also minimizing risks to public health. For example, as this paper has highlighted, suitable telehealth interventions could be devised to care for people with neurological conditions during a pandemic. In some jurisdictions, regulatory changes will be needed to enable the delivery of telehealth.

In addition, regulations could be amended to enable physiotherapists to undertake face-to-face care for their patients with neurological conditions. The regulations should also provide for adequate funding to maximize the likelihood that correct procedures (such as the use of PPE) will be implemented. During COVID-19, other health care provider, such as medical practitioners, have been lawfully permitted to provide care to patients while wearing PPE on the basis that such services are “essential.” This paper argues that physiotherapy care for people with neurological conditions is also “essential” and that governance and regulatory structures should reflect that.

This paper recognizes that developing jurisdictions may struggle to implement applicable international health law. While international health law (such as the International Health Regulations) applies to many developing countries, the implementation challenges are well-documented (58–60). For example, developing countries that are signatories of the international regulations may not have incorporated these into their domestic regulatory framework, thereby limiting the legal impact of the regulations. Furthermore, developing countries may struggle to implement regulations into their domestic health and legal systems because of limited resources and poorly designed enforcement mechanisms. Scholars have highlighted a range of strategies to address these challenges such as public-private cooperation and negotiated rulemaking (59).

Underlying regulatory structures have a strong influence on how countries are able to respond to pandemics. Overall, public health regulatory powers should allow for flexibility and the opportunity to meet not only the public health need, but also the needs of vulnerable populations.

## Conclusion

This perspective paper outlines how people with neurological conditions are a vulnerable population who may be adversely impacted by lockdown during COVID-19. It is well-known that social connection enhances health and well-being, while social disconnection is a powerful determinant of poor health and neurobiological changes. Researchers have found that the negative impacts of social isolation may be comparable to traditional clinical risk factors (61). These negative outcomes are amplified for people with neurological conditions because they need extensive care, physiotherapy, and regular activity to maintain their health. Additional magnification of these negative health outcomes occurs in jurisdictions which have poor integration of public health and social systems.

Given the potential burden on the healthcare system, and the policy focus on patient centered care, policymakers and practitioners around the globe should be keenly interested in investing in strategies to minimize the adverse health outcomes of social isolation on people with neurological conditions. Telehealth and appropriate exercise regimes have enormous potential to address the challenges, but further research is needed to provide a robust evidence base, particularly for remote assessments and interventions or hybrid models including remote and face to face components. For patients in countries with limited access to technology, including telehealth, home-based exercises provide a feasible, and accessible, alternative.

COVID-19 presents policymakers with an opportunity to recalibrate the weight given to “high needs, high cost” individuals versus public health during pandemics. With such realignment, we will be better positioned to meet the needs of people with neurological disorders and to manage the impacts of social disconnection during global pandemics.

## Data Availability Statement

The original contributions presented in the study are included in the article. Further inquiries can be directed to the corresponding author.

## Author Contributions

PP was responsible for the original idea, writing, and editing the manuscript. JS-M was responsible for writing, editing, and reviewing the manuscript. LH, CC, and SL were responsible for editing and reviewing the manuscript. All authors contributed to the article and approved the submitted version.

## Conflict of Interest

The authors declare that the research was conducted in the absence of any commercial or financial relationships that could be construed as a potential conflict of interest.
